# Effect of Morphology and Crystal Structure on the Thermal Conductivity of Titania Nanotubes

**DOI:** 10.1186/s11671-018-2613-3

**Published:** 2018-07-16

**Authors:** Saima Ali, Olli Orell, Mikko Kanerva, Simo-Pekka Hannula

**Affiliations:** 1 Department of Chemistry and Materials Science, Aalto University School of Chemical Engineering, P.O. Box 16100, FI-00076 Espoo, Finland; 2 Laboratory of Materials Science, Tampere University of Technology, P.O. Box 589, FI-33101 Tampere, Finland

**Keywords:** Thermal conductivity, Titania nanotube, Crystal structure, Rapid breakdown anodization, Chemical processing

## Abstract

Titania nanotubes (TNTs) with different morphology and crystal structure are prepared by chemical processing and rapid breakdown anodization (RBA) methods. The nanotubes are studied in terms of thermal conductivity. The TNTs with variable wall thickness below 30 nm have significantly reduced thermal conductivity than bulk titania, due to the phonon confinement, smaller phonon mean free path, and enhanced phonon boundary scattering. The amorphous nanotubes (TNT_Amor_) have comparatively thicker walls than both crystalline nanotubes. The TNT_Amor_ has a thermal conductivity of 0.98 W m^−1^ K^−1^, which is slightly less than the thermal conductivity of crystalline anatase nanotubes (TNT_A_; 1.07 W m^−1^ K^−1^). However, the titania nanotubes with mixed structure (TNT_A,T_) and the smallest dimensions have the lowest thermal conductivity of 0.75 W m^−1^ K^−1^, probably due to the phonon confinement. The experimental results are compared with the theoretical study considering the size confinement effect with different wall dimensions of TNTs and surface scattering. The results agree well with the surface roughness factor (*p*) of 0.26 for TNT_A,T_, 0.18 for TNT_A_, and 0.65 for TNT_Amor_, indicating diffusive phonon scattering and rougher surfaces for TNT_A_. Interestingly, the present results together with those presented in literature suggest that thermal conductivity reduction with respect to the wall thickness occurs also for the amorphous nanotubes. This is ascribed to the role of propagons in the thermal transport of disordered structures.

**Electronic supplementary material:**

The online version of this article (10.1186/s11671-018-2613-3) contains supplementary material, which is available to authorized users.

## Background

Due to the persistent miniaturization of the electronic devices and nano-electro-mechanical systems (NEMS), the study of nanostructures and their properties have attracted much attention in the past years [1, 2]. The studies on controlling the size and nucleation of nanostructures have been presented before, as nanostructures have been utilized for different potential applications [3, 4]. The research on controlling the thermal properties in nanostructures by controlling the size, composition, and structure is of particular interest due to their applications in the electronics industry, NEMS, and advanced thermoelectric [2, 5, 6]. One particular case is to minimize the heat dissipation in the integrated circuits (ICs) for their stability and long lifetime.

One-dimensional (1D) materials, such as carbon nanotubes (CNT), possess a room temperature thermal conductivity of 3000 W m^−1^ K^−1^, which is much higher than that of a diamond crystal [2, 5]. The CNT is a seamless rolled sheet of graphene and has higher thermal conductivity due to the strong carbon–carbon bond and no point defects and boundaries [6]. Contrary to the CNT, other one-dimensional crystalline semiconductors have significantly reduced thermal transport as compared to the bulk material [6]. This decrease in thermal conductivity in the low-dimensional nanostructures is attributed to the reduction in the phonon mean free path (MFP), small grain size, phonon boundary scattering, roughness, and point defects [6–8].

Silicon nanowires have been studied for tailoring thermal transport for their utilization in thermoelectric applications. For the first time, Li et al. [9] reported two times lower thermal conductivities for silicon nanowires compared to bulk silicon due to phonon-boundary scattering. The thermal conductivity of the silicon nanowires with the diameter of 50 nm approached the amorphous limit of silicon, with 100-fold reduction of thermal conductivity as compared to bulk silicon [10]. These silicon nanowires with considerably reduced thermal conductivity and increased electrical conductivity possess higher thermoelectric efficiency [10–13]. The reduced thermal properties of other nanowires compared to their bulk materials are also reported, such as Bi_2_Te_3_ [14, 15], Si/SiGe [16], Ge/SiGe [17, 18], ZnTe [19], GaN [20], InSb [21], CdS [22], PbS, PbSe [23], InAs [24], Bi [25], SrTiO_3_ [26], ZnO [27], and TiO_2_ nanowires [28, 29]. In addition, the thermal studies on nanotubes such as Si [30], Bi_2_Te_3_ [31], and TiO_2_ nanotubes [1, 32–34] have been reported. Based on these studies, it can be concluded that the thermal conductivity of nanotubes is less than that of the corresponding nanowires because of additional phonon scattering inside the walls of the nanotubes [31]. It should be noted the thermal conductivity of crystalline nanotubes is generally found to be higher than that of their amorphous counterparts and strongly influenced by their surface roughness [32, 34]. Furthermore, Wingert et al. [30] noticed that crystalline silicon nanotubes have lower thermal conductivity than their amorphous equivalents. This observation of thermal conductivity beyond the amorphous limit in crystalline silicon nanotubes was attributed to elastic softening and strong phonon boundary scattering [30]. The thermal transport in the amorphous nanomaterials is mainly (93%) attributed to diffusons (non-propagating “diffuson” modes), while the rest 4% is related to phonon-like modes known as “propagons” and 3% to the localized modes known as “locons” [35]. Since the mean free path of the diffusons is usually considered to be that of the interatomic distance, it is expected that the thermal conductivity of the amorphous nanostructures is independent of the dimensions [36].

Cahill and Pohl proposed a well-known minimum thermal conductivity model for the disordered materials [37]. According to that model, the proposed minimum thermal conductivity (amorphous limit) of the titania is 1.6 W m^−1^ K^−1^ [38]. No size-dependent reduction in the thermal conductivity of amorphous oxides has been reported [35] although some oxide films have been claimed to have thermal conductivity below the amorphous limit. The reason for the obtained lower value of thermal conductivity was attributed to the impurities in the structure or in the case of thin films to the thermal boundary resistance between the film and the substrate [35].

Titania nanotubes—1D nanostructures with a high specific surface area—have been designed for a number of potential applications [39]. Titania nanotubes can be synthesized by various methods including hydrothermal [40] and electrochemical anodization [39, 40], chemical processing [41], rapid breakdown anodization (RBA) [42], and template-assisted and electrospinning methods [40]. Thermal conductivity in the range of 0.40–0.84 W m^−1^ K^−1^ [1] and 0.55–0.75 W m^−1^ K^−1^ [33] have been observed for titanate nanotubes synthesized by the hydrothermal process. Brahmi et al. [32] reported a thermal conductivity of 0.85 W m^−1^ K^−1^ for a single amorphous nanotube and 1.5 W m^−1^ K^−1^ for anatase titania nanotube prepared by electrochemical anodization. On the other hand, the detached titania nanotube arrays were reported to have a thermal conductivity of 0.617 W m^−1^ K^−1^ along the tube direction for amorphous and 1.12 W m^−1^ K^−1^ for anatase nanotubes [34]. The cross-tube amorphous thermal conductivity was 0.077–0.1024 W m^−1^ K^−1^ for amorphous nanotubes and 0.24 W m^−1^ K^−1^ in the case of crystalline nanotubes [34]. Titania nanotube arrays in these reports are grown on Ti substrate by electrochemical anodization method using organic electrolytes with fluoride ions (third generation of TNTs) with a wall thickness of 30–70 nm [32] and 15 nm [34]. The nanotubes prepared by RBA comprises of the fourth generation of TNTs [43], where bundles of titania nanotubes are obtained by utilizing a fluoride-free electrolyte [42].

In the present contribution, we report a comparative experimental study on the thermal conductivity of titania nanotubes with variable morphology, crystal structure, and a wall thickness below 30 nm. The nanotubes are synthesized by chemical processing [41] and RBA [42]. The research of thermal conductivity is extended to the fourth generation of titania nanotubes (i.e., powders prepared by RBA) and to the comparison of TNT powders by different synthesis methods. Liang and Li [44] proposed an analytical model of size-dependent thermal conductivity for nanomaterials, which was confirmed experimentally for nanowires and films. The model was later modified by Gao and Jelle [1] for nanotubes but has not been experimentally verified. According to the model, the thermal conductivity of the nanotubes is dependent on the wall thickness [1]. Brahmi et al. [32] studied the thermal conductivity of TNTs with a variable wall thickness of 30–70 nm; however, reduction of thermal conductivity with the wall thickness was not observed in their study. In the present report, we experimentally verify the size-dependent thermal conductivity of titania nanotubes by reducing the wall dimensions in the crystalline titania nanotubes. Contrary to the general perception, the current data combined with those presented in the literature suggest a size-dependent reduction of thermal conductivity also for amorphous titania nanotubes.

## Methods/Experimental

### Synthesis of TNTs

**Fig. 1:**
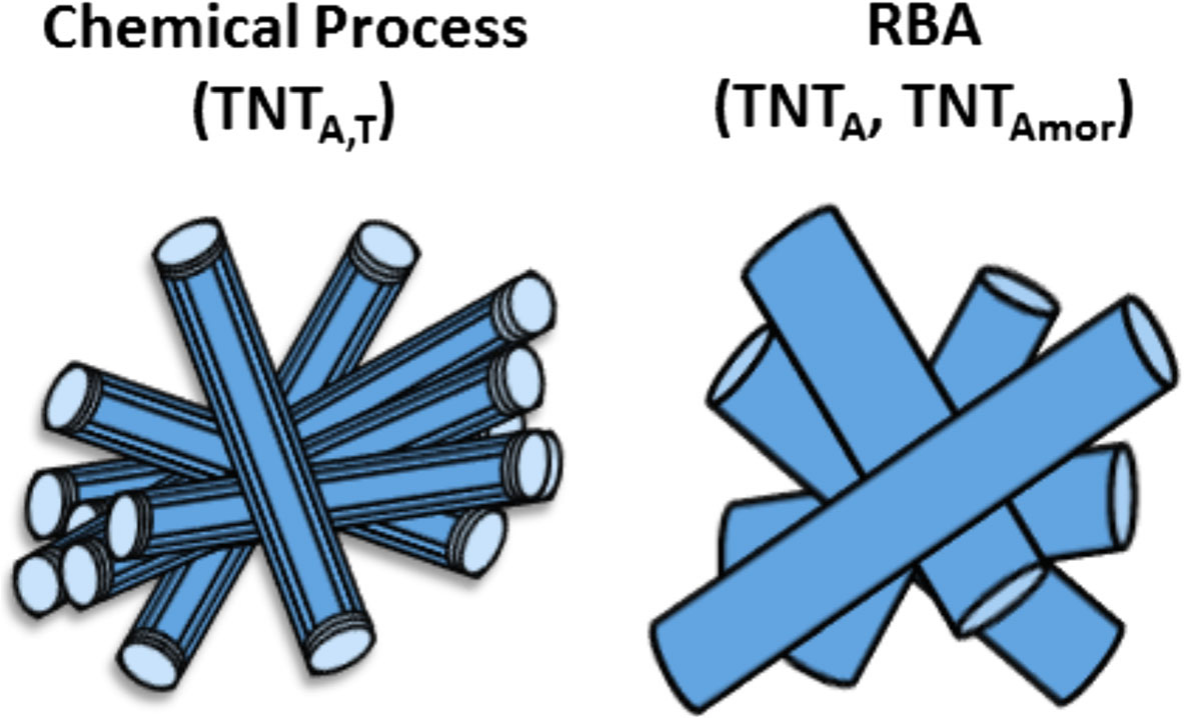
Schematic illustration of TNT_A,T_, TNT_A_, and TNT_Amor_

Titania nanotube (TNT) powders were prepared by using chemical processing and rapid breakdown anodization (RBA) methods as discussed in details in [41, 42], respectively. Three types of titania nanotubes with different crystal structure and morphology were prepared, i.e., (i) multiwalled open-ended TNTs, (ii) amorphous single-walled TNTs with one end open and the other closed, and (iii) crystalline titania nanotubes with one end open and the other one closed. The multiwalled open-ended titania nanotubes were prepared by chemical processing method and had mixed crystal structure of titanate (Na_*x*_H_2 − *x*_Ti_3_O_7_·nH_2_O, where 0 < *x* < 2) with prominent peaks from anatase phase [41] and termed as TNT_A,T_ throughout the text. Other two types of nanotubes were prepared by the RBA method either by using water-based electrolyte (0.1 M perchloric acid) to obtain crystalline TNTs with anatase structure or organic electrolyte (ethylene glycol + water + perchloric acid) to produce amorphous nanotubes [42]. The amorphous (TNT_Amor_) and crystalline (TNT_A_) titania nanotube powders produced by RBA are single-walled with one end open and the other closed. The schematic illustration of these TNTs is shown in Fig. 1.

### Characterization Methods

The morphology and size of the titania nanotube powders were examined using transmission electron microscopy (TEM; Tecnai F-20 G2 200 kV FEG S-twin GIF) at an operating voltage of 200 kV. The crystal structure was obtained by using X-ray diffraction (XRD). The XRD data was obtained by using a PANalytical X’pert Pro diffractometer. The operating wavelength was 0.154 nm Cu-Kα radiation, with the voltage and currents of 40 kV and 45 mA, respectively. The density of each powder was measured by Pycnometer (Upyc 1200e v5.04; Quantachrome Corporation). The powders were then compressed into 10-mm pellets for thermal conductivity measurements. The pellets were made by hydrostatical pressing of nanotube powders and the thickness of the pellets obtained was in the range of 2–4 mm. The measured thickness and the calculated density of the pellets are related to the applied pressure, which was controlled over a range from 5 to 50 kN to adjust each pellet’s density. The surfaces of pellets were analyzed by field emission gun scanning electron microscope (FEG-SEM; Hitachi S-4700).

Thermal diffusivity of the pellets was measured by using light flash method utilizing Netzsch LFA 467 equipment with Proteus LFA software at room temperature. A short light xenon laser pulse heated the rear surface of the pellets. Before the measurements, the pellets were coated with a graphite spray to improve the absorption and emission of the thermal radiation. An infrared detector observed the corresponding temperature change at the opposite side of the pellet. According to Parker et al. [45], the following relation can be used to obtain thermal diffusivity from the experimental data:

1 $$ \alpha =\frac{0.1338\ {d}^2}{t^{1/2}} $$

Here, *α* is the thermal diffusivity of the sample, *d* is the sample thickness, and *t*^1/2^ is the time value at the half signal height. LFA measurements were repeated for five times per sample. The Proteus software was used for fitting of the measurements. The thermal conductivity of the sample was obtained by using the following relation [45]:

2 $$ \kappa (T)=\alpha (T)\ {c}_p(T)\ \rho (T) $$

Here, *κ* denotes the thermal conductivity, *α* denotes the thermal diffusivity, *c*_*p*_ is the specific heat capacity, and *ρ* is the value of density. The specific heat capacity of titania nanotubes approaches to that of bulk titanium dioxide above 100 K [46], and therefore, the values of specific heat capacity for the titania nanotubes were adopted from a study by Guo et al. [34, 47]. The density of the pellets was calculated from the weight and the corresponding volume of the pellets. The uncertainty in the experimental results come from the errors of LFA measurement unit for diffusivity measurements (2%) and the thickness calculation of pellets by a micrometer. The total error for the thermal conductivity experiments was estimated to be 8%.

## Results and Discussion

**Fig. 2:**
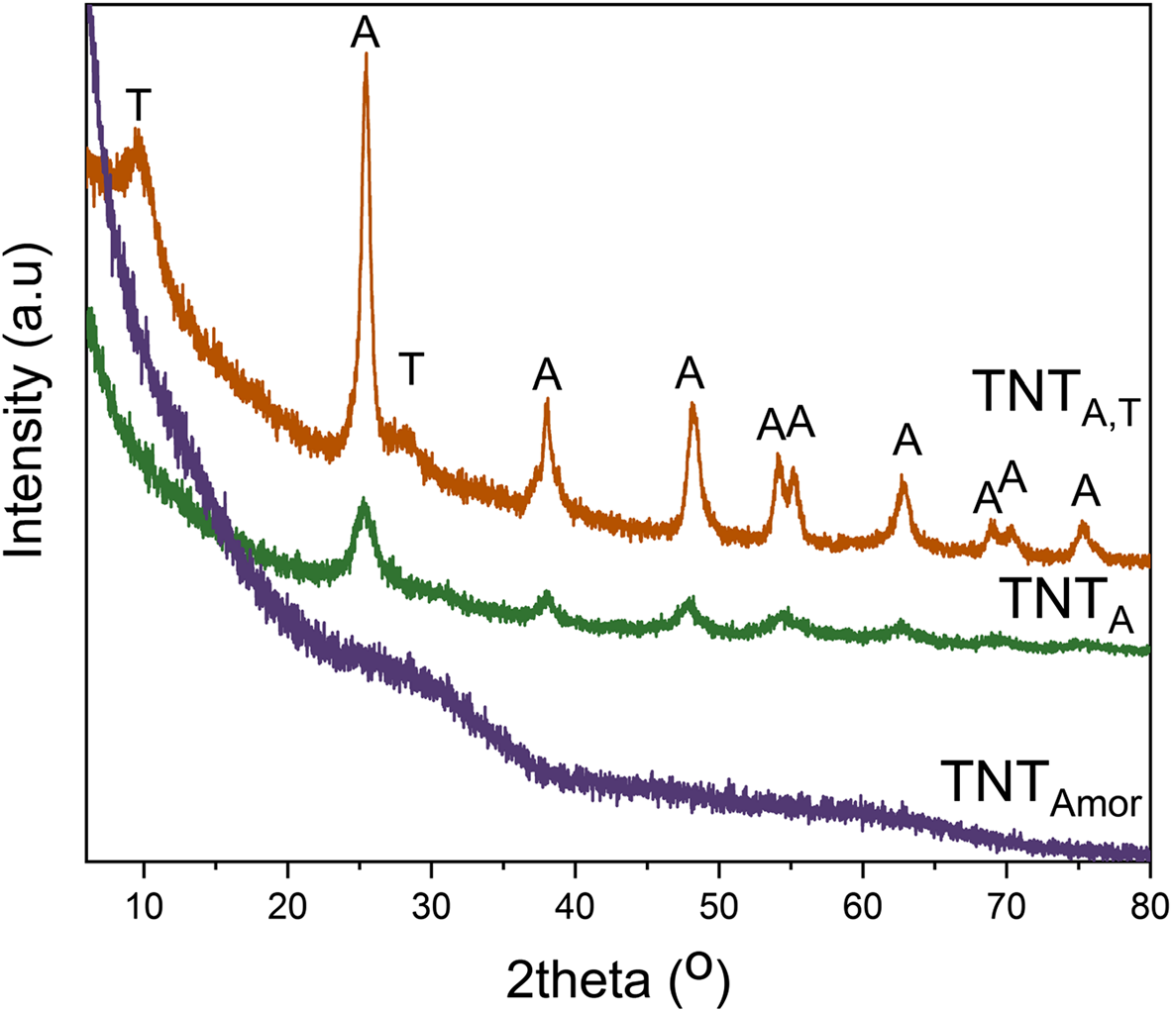
XRD of crystalline titania nanotubes consisting of anatase (TNT_A_), both titanate and anatase (TNT_A,T_), and amorphous structure (TNT_Amor_) [41, 42]. T = H_2_Ti_3_O_7_, A = anatase peaks

The XRD data for the crystal structure of the nanotubes is shown in Fig. 2. The TNT_Amor_ data has no peaks confirming the amorphous structure of the nanotubes prepared by RBA utilizing an organic electrolyte [42]. The chemically processed nanotubes (TNT_A,T_) show prominent peaks from the anatase phase along with H_2_Ti_3_O_7_ peaks. The structure other than anatase was assigned as Na_*x*_H_2 − *x*_Ti_3_O_7_·nH_2_O where 0 < *x* < 2, as reported in a previous study [41]. The TNT_A_ prepared by water-based electrolyte have anatase peaks. From the XRD data, it is obvious that two types of nanotubes are crystalline and one is amorphous.

**Fig. 3:**
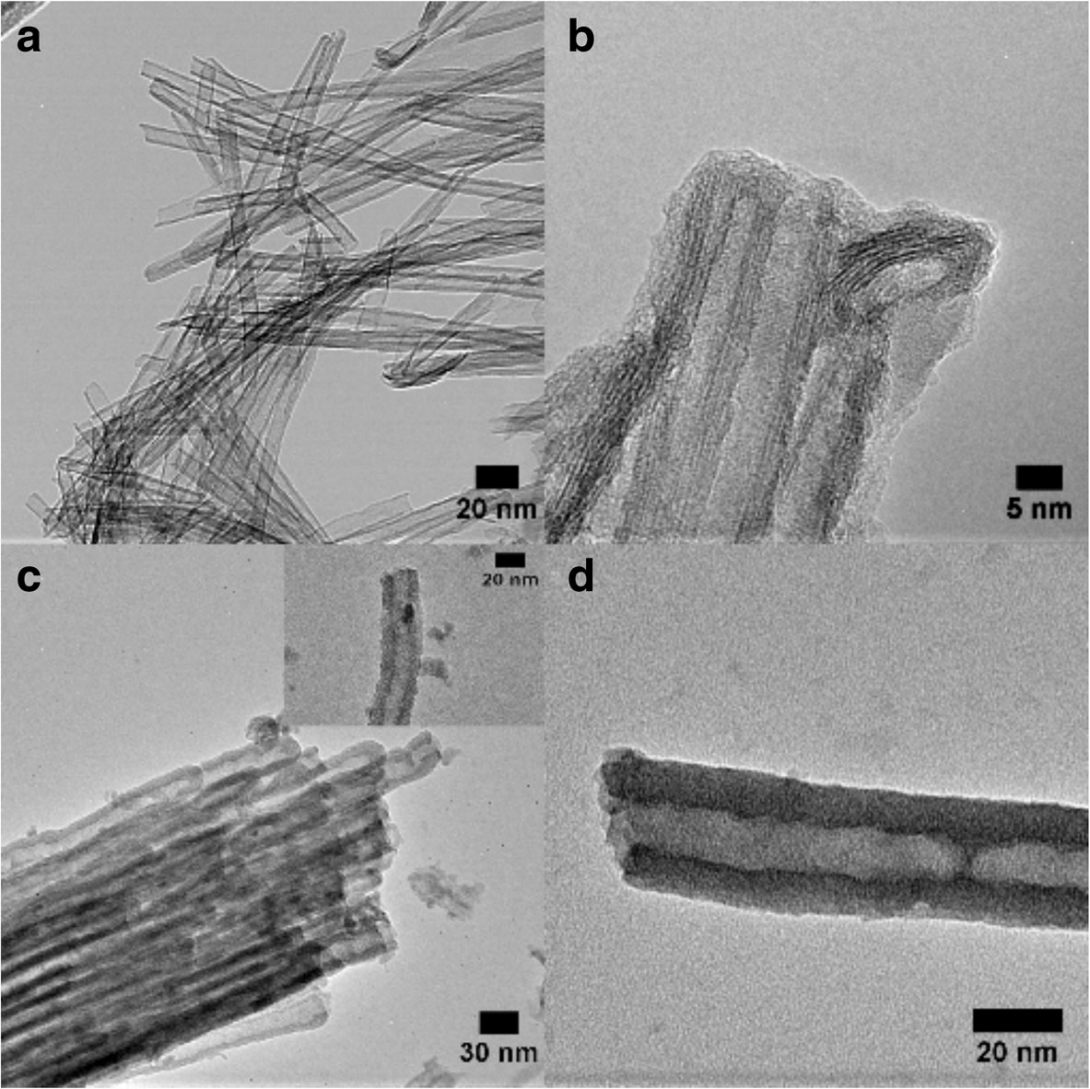
TEM images of **a** the TNT_A,T_ prepared by chemical processing, **b** HR-TEM micrograph showing the structure of multiwalled nanotubes, **c** the single-walled crystalline nanotubes prepared (TNT_A_) by RBA, and **d** the amorphous nanotubes (TNT_Amor_)

The titania nanotubes synthesized by the chemical processing method are multiwalled due to the scrolling of nanosheets during the synthesis of nanotubes [48]. These open-ended nanotubes have a wall thickness of 4–5 nm with a variable length from 60 to hundreds of nanometers [41]. TEM images from these nanotubes are shown in Fig. 3a, b. The nanotubes are randomly oriented and prefer to stay in bundles as shown in Fig. 3a. The 3- to 4-layer multiwall structure is evident as depicted in Fig. 3b. The crystalline nanotubes produced by RBA have a wall thickness in the range of 7–12 nm and are 18–35-μm long [42] (Table 1). They are single-walled with one end open and other closed as shown in the micrograph in Fig. 3c, where the inset shows the open end. The amorphous nanotubes produced by RBA have similar morphology as crystalline nanotubes prepared by the RBA method. However, the dimensions are different due to the contribution of the electrolyte. The wall thickness is in the range of 15–30 nm and the tubular length is in the range of 6–13 μm [42]. Figure 3d shows the TEM image of the single-walled amorphous nanotube. The roughness is the average value for the deviation of height of the TNT wall surface from the reference plane [44]. The average roughness values estimated from TEM images of the TNTs are approximately 0.3 nm for TNT_A,T_, 1.0 nm for TNT_A_, and 1.5 nm for TNT_Amor_.

**Table 1:** Properties of the nanotube powders used for preparing samples for the thermal conductivity studies

Sample	Crystal structure	Wall thickness (nm)	Density (g cm^−3^)	Morphology
TNT_A,T_	Anatase/ Na_*x*_H_2*x* − 1_Ti_3_O_7_·nH_2_O	4–5	3.14	Multiwalled
TNT_A_	Anatase	7–12	3.79	Single wall
TNT_Amor_	Amorphous	15–30	3.67	Single wall

**Fig. 4:**
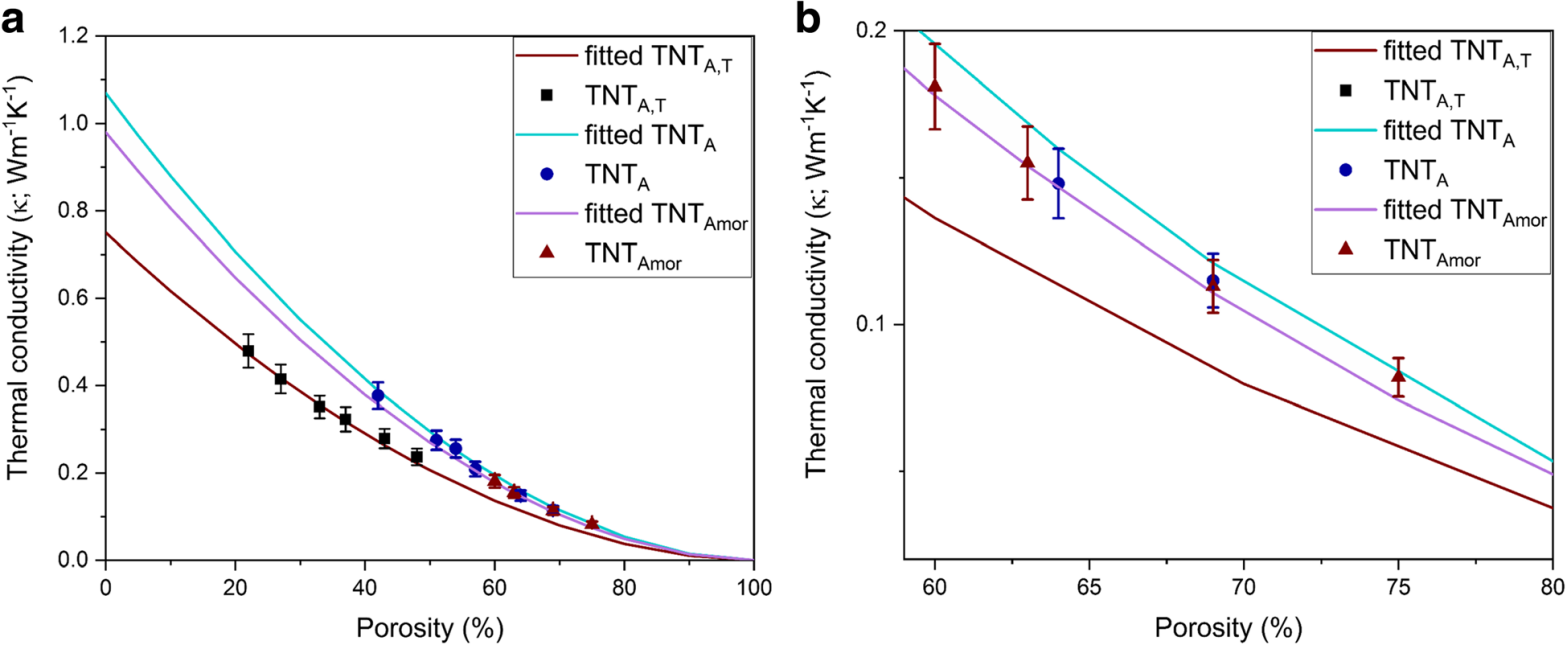
**a** Measured effective thermal conductivity of the titania nanotubes (symbols) versus porosity. The solid lines represent fitting using the effective thermal conductivity model (Eq. 6) with a shape factor of 1.24. **b** Thermal conductivity over the range of 60–80% porosity for clarity

The pellets of titania nanotubes were prepared into different densities and corresponding porosities using a hydraulic press. TNT_Amor_ powder was compacted with maximum load of 20 kN because at higher loads the smooth surface of the pellets required for the LFA measurements was not obtained. The porosity of the pellets is calculated by the following (Eq. 1):

3 $$ P=\frac{\rho_o-\rho }{\rho_o} $$

where *ρ*_o_ is the density of the bulk samples, which is the density of powder obtained by pycnometer measurements and shown in Table 1. The *ρ* is the calculated density of the pellet and *P* is the porosity of the samples. The surfaces of the pellets were studied with FESEM in Additional file 1. The analyses of the surfaces show random orientation of nanotube bundles (Additional file 1: Figure S1) on the surface, i.e., nanotubes can be observed at various orientations (open top, closed bottom, and side view positions) in Additional file 1: Figure S1. Similar SEM images of pellet surfaces from TNT_A_, TNT_Amor_, and TNT_A,T_ pellets are depicted in Additional file 1: Figure S2a–c. The measured thermal diffusivity by LFA method is summarized in Table 2. The measured thermal conductivities are plotted as a function of porosity, as shown in Fig. 4. The measured thermal conductivity decreases with increasing porosity for all the samples (Table 2). Gao and Jelle obtained a similar trend for the thermal conductivity values of samples with different porosities of pellets [1]. A clear reduction of thermal conductivity is obtained for the nanotubes compared to the bulk titania (8.5 W m^−1^ K^−1^ [34]). This suppression of thermal conductivity in 1D titania nanotubes is attributed to the phonon confinement and phonon boundary scattering due to the reduction of size [1]. As the nanotubes are randomly oriented and compacted to form pellets, they are connected to each other too. In this case, the phonon scattering at the interconnected area between the nanotubes and the Kapitza resistance also affects the overall thermal conductivity values. However, the contact Kapitza resistance and phonon boundary scattering considering the orientation of nanotubes are ignored here for simplicity.

**Table 2:** The measured thermal properties of the nanotube samples with different porosities

Samples	Porosity (%)	Thermal diffusivity (*α*) (mm^2^ s^−1^)	Thermal conductivity (*κ*_eff_) (W m^−1^ K^−1^)
TNT_A,T_	22	0.290	0.479
	27	0.260	0.415
	33	0.240	0.351
	37	0.240	0.323
	43	0.220	0.279
	48	0.210	0.237
TNT_A_	42	0.250	0.377
	51	0.215	0.275
	54	0.212	0.256
	57	0.186	0.209
	64	0.157	0.148
	69	0.141	0.115
TNT_Amor_	60	0.177	0.181
	63	0.166	0.155
	69	0.146	0.113
	75	0.128	0.082

The measured thermal conductivity of a sample estimates the conductivity of the nanotube pellets considering both the titania nanotubes and the pores filled with air. The thermal conductivity of air is presumed to be 0.026 W m^−1^ K^−1^ [1]. The thermal conductivity of the nanotubes (*κ*_TNTs_) excluding the impact of porosity can be estimated by using effective thermal conductivity models given by Eq. 4 [1, 49], which for the case of non-conducting pores reduces to Eq. 5 [1]:

4 $$ {\kappa}_{TNTs}=\frac{\upkappa_{eff}-{\upkappa}_{air}\cdot P}{\left(1-P\right)} $$

5 $$ {\kappa}_{TNTs}=\frac{\upkappa_{eff}}{\left(1-P\right)\kern0.5em } $$

where *κ*_eff_ is the effective thermal conductivity that includes the porosity effect, *κ*_air_ is the thermal conductivity of the air, and *P* is the porosity. The thermal conductivity of TNT_A,T_ estimated from Eq. 4 is in the range of 0.44–0.61 W m^−1^ K^−1^ for TNT_A,T_. Using the effective thermal conductivity model (Eq. 4), the thermal conductivity of pure titanate nanotubes with approximately similar dimensions has been reported as 0.40–0.84 W m^−1^ K^−1^ [1]. Our results agree well with the reported values when the same effective model of thermal conductivity (Eq. 4) is used.

Nevertheless, the shape of air gaps in nanotube compact is only partially random as the tubes themselves have a non-random shape. In order to account for the different shape of pores, an analytical model applicable for a full range of porosities was derived by Bauer [49] based on solving the Laplace heat conduction equation. This equation can be presented in the following form:

6 $$ \frac{\kappa_{eff}}{\kappa_{TNTs}}={\left(1-P\right)}^{\frac{3\varepsilon }{2}} $$

In this equation, *ε* is the shape factor or correction factor related to pore shape. Its value accounts for the variable shapes of the pores. For random shapes of the air gap, *ε* is 2/3 [1, 27, 50] thus reducing Eq. 5 to Eq. 6.

The values of shape factors have been estimated for polyhedral shapes by Yang et al. [50], based on modeling shape factors between 1 and 1.48. When fitting our data to Eq. 6, the best fit (see Fig. 4) is obtained for the shape factor *ε* having the value of 1.24. Based on the fit, the thermal conductivity for TNT_A_ is found to be 1.07 W m^−1^ K^−1^. This value is somewhat lower than the previously reported values, 1.12 W m^−1^ K^−1^ for anatase nanotube arrays [34] and 1.5 W m^− 1^ K^− 1^ for a single anatase nanotube [32]. Correspondingly, the thermal conductivity of TNT_Amor_ is found to be 0.98 W m^−1^ K^−1^. The slightly lower value of thermal conductivity in the amorphous nanotubes as compared to TNT_A_ is attributed to their amorphous structure. Lower thermal conductivity values of amorphous titania nanotubes than those of the crystalline nanotubes have also been reported in [32, 34]. Generally, amorphous films and materials are known to have lower thermal conductivity as compared to crystalline materials, although at such small scale other factors also influence the thermal conductivity values. For example, Wingert et al. [30] reported 30% lower thermal conductivity for the crystalline silicon nanotubes as compared to their amorphous counterparts with similar dimensions. The sub-amorphous thermal conductivity of those nanotubes was attributed to the strong elastic softening effect in the crystalline nanotubes [30]. For comparison with the amorphous films, the measured thermal conductivity of 100-nm amorphous titania film deposited by ALD process was 1.29 W m^−1^ K^−1^ [47]. The thermal conductivity approximated by the Cahill and Pohl model of the minimum thermal conductivity [37] was 1.38 W m^−1^ K^−1^ for the same film [47]. The thermal conductivity of amorphous titania films deposited by sputtering was reported to be 1.6 W m^−1^ K^−1^ for 920-nm-thick films [38, 51]. The thermal conductivity obtained for the nanotubes is smaller than that of amorphous titania films dealt with in these reports [38, 47, 51]. However, comparatively lower thermal conductivity of 0.7 W m^−1^ K^−1^ [52] was also reported for 150-nm-thick amorphous titania film prepared by sputtering and 0.9 W m^−1^ K^−1^ [53] for 120-nm-thick film prepared by sol-gel method. In the case of the films, the thermal boundary resistance between the substrate, thin film, and the metallic transducer film was considered to lower the overall thermal conductivity below the amorphous limit [52]. In case of nanotubes, factors like thermal contact resistance between the nanotubes, surface roughness, and the impurities in the structure due to the preparation process also affect the net thermal conductivity. Guo et al. [34] proposed the higher value of thermal contact resistance between amorphous nanotube arrays as compared to the crystalline nanotubes. Thermal conductivity of 0.85 W m^−1^ K^−1^ has been reported for a single amorphous nanotube [32], while Guo et al. [34] reported the thermal conductivity of 0.617 W m^−1^ K^−1^ for amorphous nanotube arrays along the tube direction. For TNT_A,T_, thermal conductivity of 0.75 W m^−1^ K^−1^ is obtained. This value agrees well with the published results for titanate nanotubes [1, 33] prepared by hydrothermal method. It is also noted that the thermal conductivity increases with the increasing density of the material shown in Table 1. The measured density of TNT_A_ (3.79 g cm^−3^) is close to the bulk anatase density of 3.89 g cm^−3^ [34]. The density of TNT_A,T_ also correlates well with the measured density of mixed titanate and titania nanostructure compacts [54]. The TNT_Amor_ has a density of 3.67 g cm^−3^, which is close to the reported density of amorphous titania film (3.73 g cm^−3^) deposited by ALD [55]. The linear dependence of thermal conductivity with density has already been reported for alumina films before [55].

**Fig. 5:**
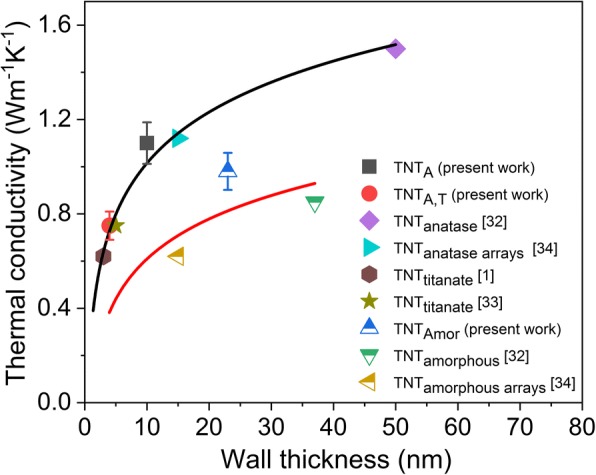
The thermal conductivity of crystalline and amorphous titania nanotubes with respect to their wall thickness. The trend lines are added for a visual guidance

The phonon mean free path has been calculated as 2.5 nm for titania [1], 1.21–3.15 nm for titania nanofibers [28], and 2–3 nm for titania nanotubes [32]. Out of the three different kinds of nanotubes studied in the present report, the anatase nanotubes (TNT_A_) yield the highest thermal conductivity value, while the thermal conductivity of multiwalled TNT_A,T_ is less than that of TNT_A_ and TNT_Amor_. Comparison of the present and previously published thermal conductivity values with respect to the wall thickness of TNTs is shown in Fig. 5. The TNTs produced from hydrothermal method, [1, 33] third-generation anodized arrays [34], and single nanotube [32], and the values from the nanotubes produced by the present RBA and chemical processing methods are plotted with their average values of wall thickness and thermal conductivities (Fig. 5). Figure 5 shows that the thermal conductivity of the crystalline titania nanotubes is reduced significantly by reducing the wall thickness. The suppression of thermal conductivity with the reduction of wall thickness is attributed to the phonon confinement with the wall thickness [32]. Although this effect was not observed by Brahmi et al. [32], obviously due to the limitation of samples with reduced dimensions, the proposed reduction is observed with the present TNT_A,T_. Figure 5 shows a similar trend for amorphous nanotubes with the reduction of thermal conductivity with the wall thickness. Generally, the amorphous nanomaterials are expected to have a similar thermal conductivity independent of the scale, as the thermal transport is attributed to the non-propagating diffusons [47]. Depending on the material and its dimensions, the propagons (propagating vibrations) may also contribute to the overall thermal conductivity [35]. Wingert et al. [35] proposed the reduction of thermal conductivity for amorphous silicon films by scaling down the film thickness from micrometer to nanometer range. Later, the size-dependent thermal conductivity reduction for amorphous silicon has been confirmed experimentally by Kwon et al. [36] due to the contribution from propagons in overall thermal transport. The mean free path of the propagons for amorphous silicon was found to be in the range of 10 nm to 10 μm and they contributed to 30% increase in thermal conductivity at the room temperature [36]. The mean free path of the amorphous titania has been estimated to be in the range of 0.195–0.201 nm (≈ interatomic distance) [56]. No study is found stating the mean free path of the propagons in titania. However, the thermal conductivity reduction with the decrease of wall thickness is also observed for amorphous TNTs (Fig. 5). It is thus speculated that the thermal transport in TNTs is ascribed not only to the diffusons, but propagons may also contribute to the overall thermal conductivity, which reduces the thermal conductivity of the amorphous nanotubes with scaling down the wall dimensions.

**Fig. 6:**
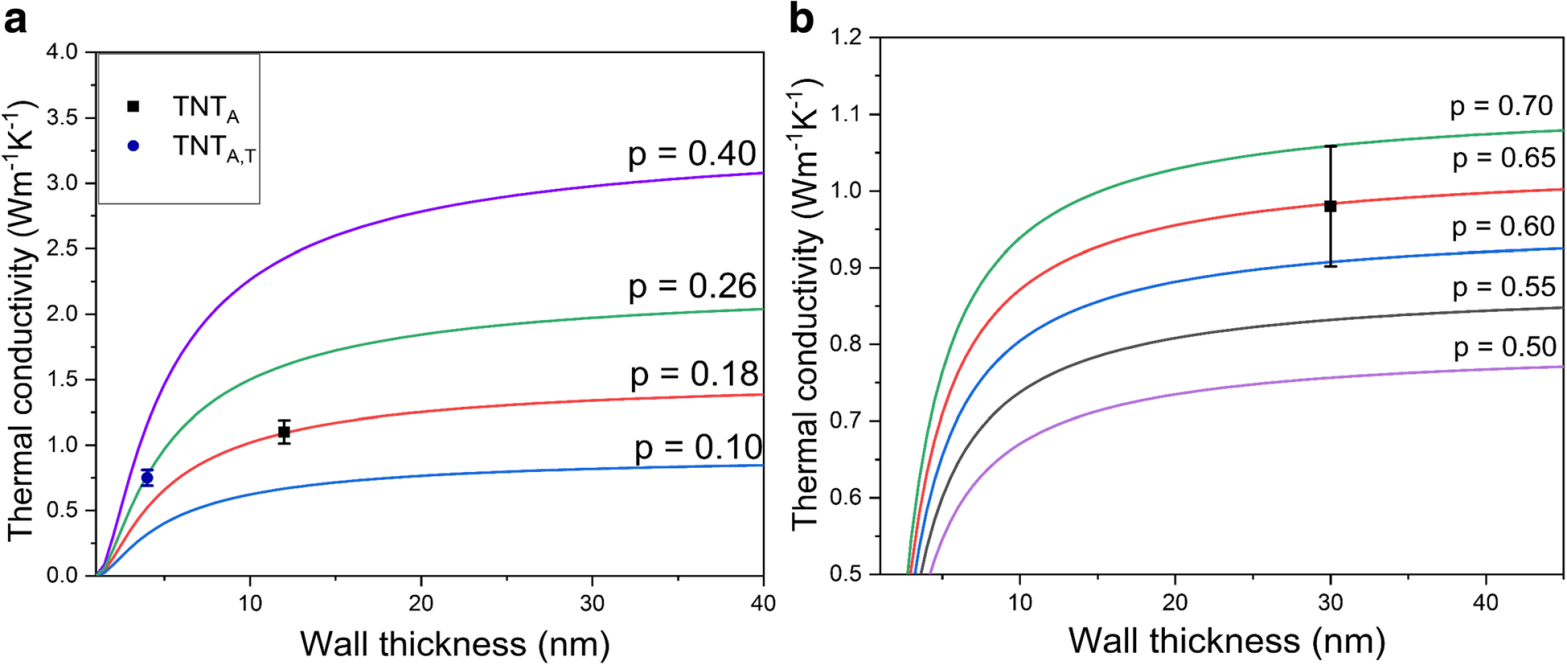
Size-dependent thermal conductivity of **a** crystalline titania nanotubes (TNT_A_ and TNT_A,T_) and **b** amorphous nanotubes (TNT_Amor_) with different surface roughness factors; symbols show the experimental thermal conductivity of the studied titania nanotubes and the solid lines indicate the calculated thermal conductivities by using Eq. 6

It has been proposed that thermal properties of the nanotubes are dependent on their wall thickness rather than the diameter [1, 32]. Gao and Jelle presented a theoretical approximation for the reduction of thermal conductivity with wall thickness [1], which was a modification of a model proposed earlier [44]. However, the overall thermal conductivity was also affected by the roughness of the nanotube surface. Liang and Li [44] proposed the analytic formula for thermal conductivity of semiconductor nanomaterial including size confinement effects, crystallinity length, and the surface scattering of phonons by the surface roughness parameter (*p*) as follows:

7 $$ \frac{\kappa_{TNT}}{\kappa_B}=p\cdot \exp \left(-\frac{l_o}{L}\right)\cdot {\left[\exp \left(\frac{1-\alpha }{\frac{L}{L_o}-1}\right)\right]}^{3/2\operatorname{}} $$

where *κ*_TNT_ is the thermal conductivity of the nanomaterial, *κ*_B_ is bulk thermal conductivity, *l*_o_ is the phonon mean free path, *L* is the wall thickness, and *L*_o_ is the critical size at which almost all atoms of a crystal are located on its surface [44]. It should be noted that *L*_o_ = 2(3 − *d*)*w*, where *d* is the dimension of the material (which is 1 in the case of nanotubes) and *w* is the atomic or molecular diameter [1, 44]. Finally, *α* is a material constant = 2*Sv*/3*R +* 1, where *Sv* is the bulk vibrational entropy and *R* is the ideal gas constant [44]. The phonon mean free path of the titania nanotubes calculated from the kinetic formula of lattice thermal conductivity was reported to be 2.5 nm [1]. The bulk thermal conductivity of titania (*κ*_B_) is 8.5 W m^−1^ K^−1^ as noted previously. The values for *w*, *Sv*, and *α* are obtained from the study by Gao and Jelle [1]. The surface roughness factor *p* obtains values from 0 to 1, where smaller value of *p* corresponds to a rougher surface and diffusive phonon scattering and larger values correspond to smooth surfaces with specular phonon scattering [1, 32, 44]. Figure 6a shows the thermal conductivities of crystalline nanotubes for different wall thicknesses and scattering factors. The *p* factor of 0.4 was found best for estimating the thermal conductivity of 2-nm rutile nanoparticles in [57] as well as for silicon nanowires having the diameter of 20–100 nm in [44]. The same *p* value of 0.4 has also been used for titanate nanotubes by Gao and Jelle [1], who theoretically estimated thermal conductivity values of TNTs between 0.30 and 0.77 W m^−1^ K^−1^ for 2–3-nm wall thickness. Contrary to the previous reports, by using Eq. 7 our experimental data for TNT_A,T_ fit with the *p* factor of 0.26 as shown in Fig. 6a. The practical value is plotted at a maximum wall thickness. For TNT_A_, the thermal conductivity value obtained by using Eq. 7 at the maximum wall thickness (12 nm) fits with the calculated surface roughness factor of 0.18. These small values are associated with the rough surface of the anodized nanotubes. The *p* factor corresponds to *p =* 1 − 10*η*/*L*, where *η* is the surface roughness of nanotubes and *L* is the thickness of the material [44]. This equation gives the approximation of surface roughness of 0.22–0.29 nm for TNT_A,T_ and 0.56–0.96 nm for TNT_A_. These values correlate quite well with the roughness values estimated from the TEM micrographs. The difference in surface roughness for both nanotubes results from the synthesis process. It is pointed out that the thermal conductivity increases with increasing wall thickness for both crystalline nanotubes. This provides experimental verification for the model proposed by Liang and Li [44] and modified for nanotubes by Gao and Jelle [1], where thermal conductivity increases with an increase in wall thickness. The decline in the wall dimensions leads to the reduced phonon mean free path by phonon confinement and increased diffuse phonon boundary scattering, resulting in overall reduction in thermal conductivity values [32]. The crystal defects as well should influence the net thermal conductivity value along with the thermal contact resistance between the nanotubes, which are not considered here. Equation 7 is also adapted for the amorphous nanotubes (TNT_Amor_) and the maximum value of wall thickness (30 nm) is plotted in Fig. 6b. The bulk thermal conductivity (*κ*_B_) of the titania is estimated as 1.6 W m^−1^ K^−1^ [38] from the minimum thermal conductivity model and *l*_o_ is estimated as 0.198 nm [56]. The experimental value fits well with the *p* factor of 0.65 for amorphous nanotubes, which gives the surface roughness of 0.99–1.98 nm for the TNT_Amor_. The mean roughness of TNT_Amor_ estimated from the TEM images (1.5 nm) fits well with this theoretical range. The surface roughness in one-dimensional crystalline nanostructures (< 100 nm) has a strong impact on the overall thermal conductivity reduction due to the diffusive phonon boundary scattering [58, 59]. In the case of amorphous material, the surface roughness could play a role if it approaches the wavelength of the propagons [36].

## Conclusions

Three different kinds of titania nanotubes are synthesized with different crystal structure and morphology by using chemical processing and rapid breakdown anodization methods. Based on the measurement results at room temperature, the thermal conductivity of the titania nanotubes is considerably lower as compared to the bulk titania. Titania (TNT_A_) nanotubes are single-walled with one end opened and other closed, and they have anatase structure and a wall thickness of 7–12 nm. The thermal conductivity of these nanotubes estimated by an effective model of thermal conductivity is 1.07 W m^−1^ K^−1^. The amorphous nanotubes (TNT_Amor_) with a wall thickness of 15–30 nm have a thermal conductivity of 0.98 W m^−1^ K^−1^. Their thermal conductivity is slightly lower than that of crystalline anatase nanotubes (TNT_A_). However, the multiwalled and open-ended nanotubes (TNT_A,T_) with a mixed crystal structure and a wall thickness of 4–5 nm have the lowest thermal conductivity of 0.75 W m^−1^ K^−1^. This low value of thermal conductivity is due to the reduced dimensions of walls approaching the calculated 2.5-nm phonon mean free path. The reduction in the wall thickness is found to result in overall suppression of the thermal conductivity as the phonon confinement is enhanced and the phonon boundary scattering increased. The size confinement effects of phonon transport with different surface-related parameters for both crystalline and amorphous nanotubes are considered. Generally, the thermal conductivity of amorphous oxides is found independent of the size. Comparison of the present result on the amorphous nanotubes with those in the literature, however, suggests also size-dependent reduction in the thermal conductivity of the amorphous nanotubes. This may be due to the possible contribution of propagons in the overall thermal transport in disordered structure along with the diffusons. For TNT_A,T_, the thermal conductivity value agrees well with the surface roughness factor of 0.26, while in the case of TNT_A_ nanotubes, it matches with 0.18 confirming the different surface roughness of the two kinds of crystalline nanotubes related to the synthesis processes. TNT_Amor_ surface roughness (1.5 nm) estimated from TEM micrographs is in line with the calculated surface roughness factor of 0.65.

## Additional File

Additional file 1: **Figure S1.** SEM image from TNT_Amor_ pellet showing the random orientation of nanotube bundles. Figure S2 The SEM image from the surface of pellets; a TNT_A_, b TNT_Amor_, c TNT_A,T_. (DOCX 236 kb)

## Abbreviations

RBA: Rapid breakdown anodization
SEM: Scanning electron microscopy
TEM: Transmission electron microscopy
TNT_A_: Titania nanotubes with anatase crystal structure
TNT_A,T_: Titania nanotubes with mixed crystal structure (anatase and titanate)
TNT_Amor_: Titania nanotubes with amorphous structure
TNTs: Titania nanotubes
XRD: X-ray diffraction
