# Supplementary material for: Effect of Morphology and Crystal Structure on the Thermal Conductivity of Titania Nanotubes
Source: Nanoscale Res Lett. 2018 Jul 16;13:212. doi: 10.1186/s11671-018-2613-3 (PMC6047950; doi:10.1186/s11671-018-2613-3)
Supplement: Supplementary file 1 — Figure S1. SEM image from TNTAmor pellet showing the random orientation of nanotube bundles. Figure S2 The SEM image from the surface of pellets; a TNTA, b TNTAmor, c TNTA,T. (DOCX 236 kb) [file 11671_2018_2613_MOESM1_ESM.docx]

Additional File

**Effect of morphology and crystal structure on the thermal conductivity of titania nanotubes**

**Saima Ali ^1,^*, Olli Orell^2^, Mikko Kanerva^2^ and Simo-Pekka Hannula^1^**

^1^Department of Chemistry and Materials Science, Aalto University School of Chemical Engineering, P.O. Box 16100, Espoo FI-00076,Finland

^2^Tampere University of Technology, Laboratory of Materials Science, P.O. Box 589, Tampere FI-33101, Finland

*Corresponding author

**Email:** [*****saima.ali@aalto.fi](mailto:*saima.ali@aalto.fi); [olli.orell@aalto.fi](mailto:olli.orell@aalto.fi); [mikko.kanerva@tut.fi](mailto:mikko.kanerva@tut.fi); [simo-pekka.hannula@aalto.fi](mailto:simo-pekka.hannula@aalto.fi)



Results and Discussion

**Figure S1** SEM image from TNT_Amor_ pellet showing the random orientation of nanotube bundles





**Figure S2** The SEM image from the surface of pellets; **a** TNT_A_, **b** TNT_Amor_ **c** TNT_A,T_
